# Comparing Cooperative Flipped Learning with Individual Flipped Learning in a Biochemistry Course

**DOI:** 10.25122/jml-2019-0149

**Published:** 2020

**Authors:** Fatemeh Jafarkhani, Zahra Jamebozorg

**Affiliations:** Department of Educational Technology, Faculty of Psychology and Educational Sciences,Allameh Tabataba’i University, Tehran, Iran

**Keywords:** Blended approach, flipped learning, group teaching, individual teaching

## Abstract

The aim of this study was to compare a cooperative flipped learning method with an individual flipped learning method in a biochemistry course. In a quasi-experiment, convenience sampling was employed to select 61 biochemistry students, who were then randomly divided into three groups. The experimental groups were trained separately using the cooperative flipped learning and individual flipped learning methods in seven sessions. The control group was trained using the conventional method. The groups were given teacher-made academic achievement tests, used as pretest and posttest. The ANCOVA test was employed to analyze data. According to the results, the cooperative flipped learning method improved academic performance more than the individual flipped learning method in the posttest scores. The results also indicated that the flipped learning implementation both outside and inside the classroom has effects on learning. In fact, the optimal implementation of flipped learning improves in-depth learning to solve problems and carry out experiments in a biochemistry course.

## Introduction

Blended learning focuses mainly on learner engagement, which is better provided by the internet and information communication technology. The blended learning approach is based on the thoughtful combination of individual training and online training, reconsideration of curriculum design for the highest possible level of learning, and restoration and replacement of traditional class hours [1]. Bergmann and Sams formulated specific policies on creating a self-sustaining learning community in the classroom to encourage learners in self-regulation in accordance with the flipped learning technique [2]. Some of such measures include participating in workgroups, making groups that develop the student-centered class culture, promoting guided metacognition, introducing new activities, respecting personal differences, trusting learners, and supporting their failures by providing appropriate opportunities. The flipped learning method is student-centered, which benefits from certain techniques such as exploration, discovery, and group activities [3]. Cooperative learning is possible when learners help each other achieve the learning goal in a group through discussion and a question-and-answer process. In fact, this type of learning is based on the intergroup social relationship [4]. If learners are involved in cooperative learning, they make more effort to fulfill their goals, in comparison with the learners who learn alone [5]. Biochemistry is an essential basic course taken by medical science students. Learner competence of formula calculation and balance, and also the person’s interactions and metabolism have a key role in improving their self-confidence.

Learners should improve this particular skill so that they can increase their responsiveness speed in the analysis and calculation of biochemistry exercises. Due to the great importance of this skill, a significant part of the course syllabus has been allocated to it. However, the limited class time does not allow learners to do enough exercises. On the other hand, the instructor is not present to help them perceive problems correctly so that they do not lose motivation. Therefore, modern technology-based teaching methods can serve as an appropriate strategy. The flipped learning technique is a method in which teachers provide students with the teaching material prior to a class so that they can be taught the concepts of new information before the class is held. However, relevant and supplementary assignments, exercises, and problems are solved in the class. In fact, students save time that they usually spend writing down sample problems or taking notes. Instead, there is enough time to provide support and get feedback on learning goals. Reportedly, the flipped learning technique has a very diverse research background. This method has recently been used in different areas such as science [6], computers [7], health education [8], mathematics [9, 10] statistics [11], and engineering [12]. 

Previous studies unanimously indicated that the flipped learning technique has positive effects on learning and learner motivation. On the other hand, there are different implementation methods of this technique both outside the classroom (watching movies, doing assignments, and reading texts) and inside the classroom (discussion, personal or group presentation, short quizzes, and roleplaying) [13]. The aforementioned methods appear to be in the initial stages of development. In other words, there are no explicit models as to how to implement the flipped learning technique best. This study aimed to compare the cooperative flipped learning technique with the individual flipped learning technique in solving biochemistry exercises. In fact, the research subject was to determine whether there was a difference between the scores of the students trained with the individual flipped learning technique and those trained using the cooperative learning technique. Therefore, the research hypothesis stated that there is a difference between the cooperative flipped learning technique and the individual classroom technique in a biochemistry course.

## Material and Methods

This study is a pretest-posttest quasi-experiment with a control group. The independent variables were the cooperative and individual flipped learning techniques, whereas the dependent variable was the learning rate. Since the instructor of the sections was the same, he formed the new three groups by systematic random assignment. Then, the new groups were also randomly assigned to the control and experimental groups. 

The statistical sample included 61 medical sciences students (41 females and 20 males) in the academic year 2015-2016 that took place in the context of a nursing undergraduate ﬁrst course of “biochemistry” titled “metabolism of carbohydrates and lipids”. The students were expected to explain the molecular structure of carbohydrates and lipids, as well as their classification and the interactions that take place in the body. The gender distribution was not equal, as the female students outnumbered male students in all the groups. The three classes were taught by the same instructor using the same course material. Before the implementation, all the participants agreed upon the study research according to the ethical standards of the university guidelines. 

At first, a pretest was conducted for the three groups. The students’ grades (learning rate) were obtained from the in-class quizzes and ﬁnal exam scores. In fact, the posttest grade of each student is calculated from the mean weight of quizzes and the ﬁnal exam result at the end of 6 weeks. While the students were assigned to the three groups by systematic random sampling, the experimental groups and control group were also determined randomly. 

In the first group, the activities would be performed cooperatively in the class, the second group would perform activities individually in the class, and the control group would perform activities at home as per the standard method. In the experimental group using the cooperative technique, the teams were designed with at least three students whose levels were decided according to their entrances exam scores, which means that each team includes students with low, middle and high grades. A web-based learning management system was used to present video contents, course materials, and test construction activities. The students were directed to login by using the university ID cards by different devices at any time or anywhere. 

During the implementation, each student in both experimental groups watched the videos or read the materials such as notes or PowerPoint slides presented, and after that, they started working on the questions and exercises before they came to the class. Then they were asked to complete a quiz before participating in the in-class activities to ensure whether they watched the videos, read the materials, or did the exercises. In the control group, the instructor taught the contents of each session in the class using the question and answer method, along with a lecture accompanied by practical exercises. Then, the instructor wanted the students to complete the activities at home. In the next session, the ambiguities of the exercises were explained. The necessary information was collected through tests. Pretests, quizzes, and posttests were designed with the same questions of equal difficulty level for each group, which were presented in descriptive, short-answer, multiple-choice, and fill-in-the-blank forms. The validity of the questions was confirmed by five biochemistry professors. 

The collected data were analyzed using SPSS at two levels. The measures of central tendency were used for the descriptive statistics, and the ANCOVA test was employed for the inferential statistics in order to investigate if there is a significant difference between means in the three models of teaching. Based on the pretest scores and university entrance exam scores, the readiness levels of the groups were controlled, and no significant difference was observed. 

## Results

The results are reported in two sections: first, descriptive statistics were reported, and then inferential statistics regarding the research question were presented. Table 1 shows the descriptive indicators of the three research groups by test type.

**Table 1:** Descriptive statistics in the three groups.

**Group**	**Test**	**Freq**	**Mean**	**SD**	**Skew**	**Std E S**	**Kur**	**Std E K**	**Min**	**Max**
**Control**	Pretest	20	3.24	1.37	0.16	0.51	-0.49	0.99	1	10
	Postest	20	12.75	1.92	-0.05	0.51	-1.28	0.99	6	16
**Cooperative flipped**	Pretest	20	3.56	1.34	-0.09	0.51	-0.72	0.99	1	10.50
	Postest	20	15.71	2.41	-0.60	2.41	-0.60	2.41		
**Individual flipped**	Pretest	20	4.10	1.34	0.13	0.51	-1.25	0.99	1	6
	Postest	20	13.38	1.38	0.31	0.51	-0.37	0.99	11	16

Note: Freq: Frequency, Skew: Skewness, Std E S: Standard Error of Skewness, Kur: Kurtosis, Std E K: Standard Error of Kurtosis.

Before the interpretation of ANCOVA output, the assumptions related to it, the sample size, normality and outliers, linearity, multicollinearity and singularity, and homogeneity of variance/covariance matrices on the dependent variable, were checked. No imbalance was observed.

Table 2 indicates the results based on the main analysis to see if there is any difference between the cooperative flipped learning technique and the individual classroom technique. 

**Table 2:** Tests of between-subjects effects.

**Source**	**Sum of Squ.**	**df**	**Mean Squ.**	**F**	**Sig**
**Corrected Model**	90.71	3	30.23	7.85	0.001
**Intercept**	1419.25	1	1419.25	368.38	
**Pretest**	0.32	1	0.32	0.08	0.77
**Group**	90.71	2	3.36	11.77	0.001
**Error**	215.75	56			
**Total**	12206.86	60			
**Corrected total**	306.49	59			

Accordingly, the variable was significant at 0.01 (F=11.77, df=2). In other words, the methods have been effective in the flipped learning groups compared to the control group. The Bonferroni post-hoc test was used to determine on which group the method had the greatest effect.

The results of Table 3 show the superiority of the cooperative group over the individual group. Furthermore, the results of Table 1 show a significant difference between the mean of the control group (12.75) and that of the training group (15.71). The difference shows the superiority of the cooperative flipped learning technique. There was also a significant difference between the mean of cooperative flipped learning technique (15.71), and that of the individual flipped learning technique (13.38). Consequently, the cooperative flipped learning technique had the highest mean.

**Table 3:** Bonferroni Comparison.

**Comparison**	**Mean Differences**	**Std. Error**	**Sig**	**Confidence Interval**	
				**Upper bound**	**Lower bound**
**Group**	-2.98	0.62	0.001	-4.52	-1.44
**Individual**	1.90	0.63	0.01	0.34	3.45

## Discussion

The flipped learning model is gaining popularity as a blended approach to promote learner-centered education in a wide range of educational arrangements. The aim of this study was to compare the effects of the flipped learning method cooperatively with learning it individually in a biochemistry course. There was a signiﬁcant difference in the learning level of the flipped cooperative group and individual group. The results were also consistent with the findings of Chen [14], DeLozier and Rhodes [15], Foldnes [16], Kirschner et al. [17], Gadgil et al. [18]. Prerecorded videos were provided for learners to help them learn and perceive educational content faster and better. This preparation stimulates the curiosity of learners so that they can understand what they do not know and what they ought to know. 

Therefore, the class time in both experimental groups is spent solving problems in the presence of teachers so that every enthusiastic student can ask questions resulting in more detailed learning and preventing them from practicing fruitless memorization. In fact, educational design affects other consequences with respect to different audiences. During the class activities, the professor acted as a facilitator or guide to encourage learners to work individually or cooperatively to learn better. But concerning cooperative working, the students have more opportunities to develop new learning strategies that can not be achieved in an individual flipped learning or in normal traditional classes. The group members are not homogeneous, so there is a growing discussion in order to convince each other since there is one goal for all, and they would share their understandings. Then in such an environment, higher-level thinking and a sense of altruism instead of self-satisfaction are formed. On the other hand, when creative discussions take place, new and creative solutions flourish. Finally, the right leadership of the instructor will lead to deep learning. More importantly, when students are satisfied with their achievements, they feel happy and motivated. 

Another important point to note is the learner’s attitude to cooperative working and how they feel having the possibility to help their classmates. The results of this study were not consistent with the findings of Eryilmaz and Cigdemoglu [19], Seyedmonir, Barry, and Seyedmonir [20]. Depending on the different attitudes or personal growth as well as the field or type of education, we may encounter different results. 

After the implementation of the project, the students claimed in an interview that if there was not a quiz at the beginning of each session, they might not watch the videos completely or follow the learning management system instructions. It was also revealed that introvert students were more satisfied with the individual flipped class. Some of the students noted that they do not like spending time on watching videos or reading articles before the class, which reveals some defects in deep learning and study methods of the students while the instructor was satisfied with his role since he had more time to observe the students and assess their work. However, based on the interview results, most of the students enjoyed the possibility to work together and solve the doubts they had. Moreover, they were most satisfied with their deep learning so that they would be ready for the final exam with a short review on the night before the exam, claiming that it would help them save time for other lessons. 

The findings of the present study contribute to medical sciences education from some perspectives. Since medical students have practical lessons in addition to theoretical courses, implementing the flipped learning technique provides them a better way to save time as well as deep learning. It also showed that they need some guidance for better study methods to understand the importance of pre-reading lessons and improve their ability in self-regulated behaviors. Paying attention to individuals’ personality styles is another important point in performing different techniques of flipped learning, especially in groups or individually. However, the effect of cooperative learning depends on different factors such as lesson complexity, relevant experiences of learners, and learner commitment to the group. Due to the positive effect and the increased positive capacity, certain measures such as leadership skills, improved teamwork capability, and social supports for such activities must be noted. Perhaps, many of such skills are required not only in education but also in everyday life. It was also revealed that the pedagogical advantages of flipped learning might be affected in terms of field study varieties or students’ genre.

## Conclusion

This study highlighted the pedagogical contributions of the flipped learning techniques, such as fostering autonomy, collaboration opportunities, as well as the importance of in-class activities in higher education. This study was conducted on a group of students in a biochemistry course. However, it is still necessary to carry out more studies in order to find the best implementation of flipped learning techniques for medical students or other fields of study. Furthermore, different implementation methods can be considered for different age groups among school or university students using cooperative, roleplaying, simulations, or problem-solving techniques. Since most of the sample were female students, the outcomes could be affected, so further research is recommended on gender differences. The number of students in the sample might be another limitation to the research. In future studies, a higher number of students may lead to better results and generalization. 

## Conflict of Interest

The authors declare that there is no conflict of interest.
